# Idiopathic Small Bowel Intussusception in Adults: Conservative Versus Surgical Management

**DOI:** 10.7759/cureus.45460

**Published:** 2023-09-18

**Authors:** Rachel Pindek, Ethan Shamsian, Molly Mcdonald, Abralena Wilson, Kevin Louie

**Affiliations:** 1 Medicine, New York Institute of Technology College of Osteopathic Medicine, Old Westbury, USA; 2 Surgery, Mount Sinai South Nassau, Oceanside, USA; 3 Acute Care Surgery, Mercy Hospital, Rockville Centre, USA

**Keywords:** intussusception, idiopathic adult intussusception, idiopathic intussusception, small bowel intussusception, general surgery

## Abstract

Intussusception is a rare diagnosis in adults and generally has a pathological cause. In this case report, we highlight an adult, male patient who presented with typical signs and symptoms of intussusception, and a diagnosis was subsequently confirmed with imaging. After 24 hours of no clinical improvement, the intussusception was resolved through a laparoscopic approach. No lead point or other pathological cause was identified that may have contributed to the development of intussusception. The idiopathic presentation of intussusception in adults is scarcely represented in the scientific literature, making its best management practices vague and leaving room for studies regarding best surgical management. We conducted a brief literature review of adult idiopathic intussusception cases and found fewer than 25 cases documented since 2010. Our analysis revealed that the majority of cases were resolved through a laparoscopic method and only about a quarter were conservatively managed with supportive measures. More research is needed in this subject matter to more accurately determine the need for surgical management in cases of adult idiopathic intussusception.

## Introduction

Intussusception is the “telescoping” of one segment of the intestine into an adjacent segment. It can be grouped into the following categories based on the location of the telescoping bowel: (i) entero-enteric, (ii) colo-colic, (iii) ileo-colic, and (iv) ileo-cecal. Intussusception is commonly seen in the pediatric population, presenting with intermittent, crampy abdominal pain, nausea, and bilious vomiting. Later symptoms may include “red currant jelly” stool, and examination may reveal a “sausage-shaped” mass on palpation of the abdomen [1]. Factors that have been implicated in the development of intussusception in the pediatric population include infection leading to hyperplasia of Peyer’s patches, or an anatomical “lead point” such as a Meckel’s diverticulum, which is found in only about 10% of cases [2]. Most cases of intussusception in pediatric patients are of unknown cause.

In contrast, intussusception in adults is exceedingly rare accounting for 1-5% of mechanical bowel obstructions [1,2], and associated with a pathological cause in almost 90% of symptomatic cases [1]. The most common presenting symptoms in adults are abdominal pain [1] and nausea. Diagnosis is often made using abdominal CT, which shows a “target sign” of a telescoping bowel on sagittal view, or a sausage-shaped mass on coronal/axial view. Currently, there is no general consensus on how to treat adult intussusception. Historically, adults with colonic intussusception have undergone resection en bloc due to the high probability of malignancy, with pathologic lead points being found in up to 66% of cases [1]. However, in cases of small bowel intussusception, surgery is perhaps not the best approach as a majority of cases are non-pathologic, with only around 30% due to malignancy [1].

Whether non-operative treatment should first be considered in regard to small bowel intussusception is yet to be explored. Interestingly, there has been an increase in cases of idiopathic adult intussusception published recently. Here, we present a case report of idiopathic adult entero-enteric intussusception that was managed operatively.

## Case presentation

**Figure 1:**
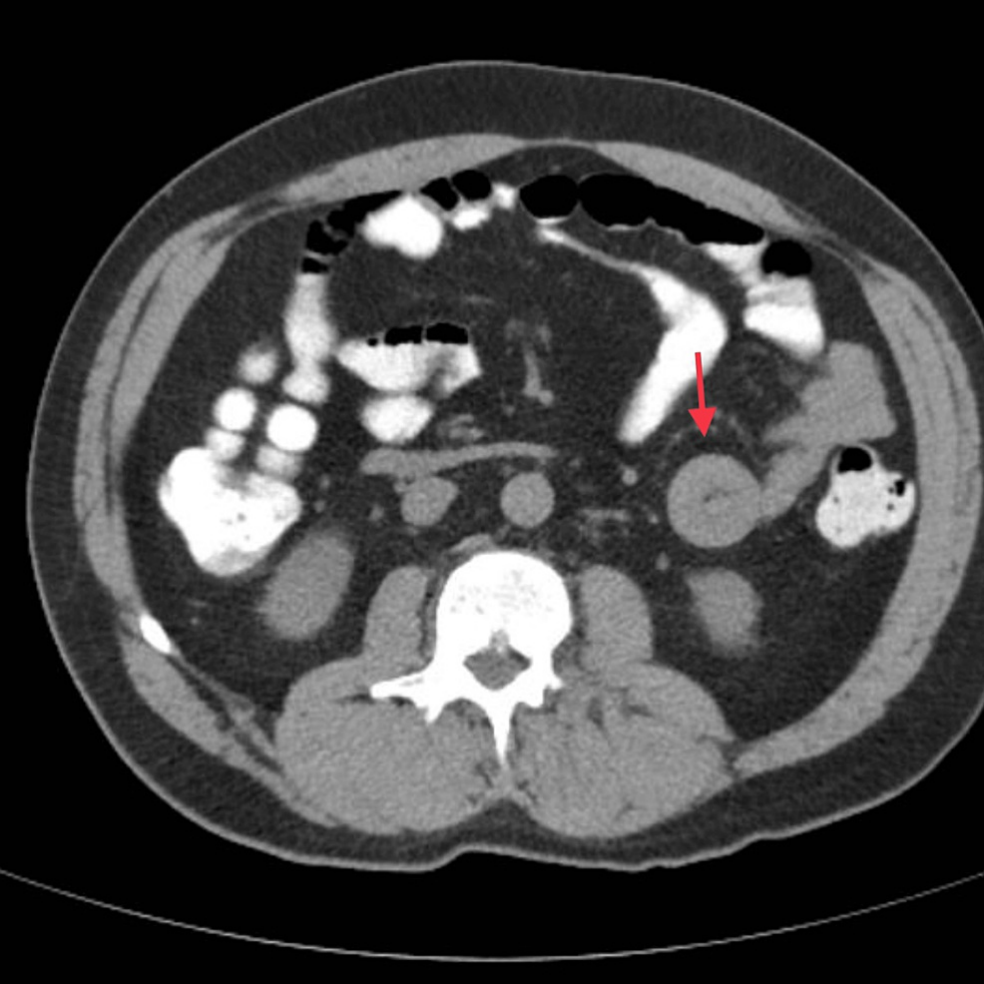
CT scan demonstrating the classical “target” sign morphology of intussusception.

A 48-year-old male presented to the emergency room with a two-day history of left-sided abdominal pain described as dull, rated 4/10, worse on palpation, and associated with nausea, chills, and reported fevers. The patient reported flatus but had not had a bowel movement in two days. A review of the systems was negative for vomiting, diarrhea, hematuria, and dysuria. Past medical history was significant for gastritis, gastroesophageal reflux disease, hyperlipidemia, and hemochromatosis. No past surgical history was reported. Recent colonoscopy and esophagoduodenoscopy performed before this hospital stay were unremarkable. Vital signs were within normal limits. On the physical examination, there was tenderness to palpation in the left lower quadrant, with no rigidity or guarding. Clinical laboratory tests were unremarkable except for blood urea nitrogen of 19 mg/dL (7-18 mg/dL), creatinine of 1.50 mg/dL (0.70-1.30 mg/dL), estimated glomerular filtration rate of 57 L (≥90 mL/minute), and a white blood cell count of 3.8 x 10^3^/µL (3.30 - 10.80 x 10^3^/µL). All other studies were within normal limits. A CT of the abdomen and pelvis without contrast was performed and revealed a short segment of small bowel-small bowel intussusception in the left hemiabdomen with no obstruction. A repeat CT of the abdomen and pelvis with oral contrast confirmed the diagnosis of intussusception (Figure 1). A third CT was performed 24 hours later which showed persistent intussusception.

Given the patient’s persistent intussusception >24 hours and no improvement in pain, the decision was made to proceed with operative exploration. The patient was taken to the operating room for diagnostic laparoscopy, which revealed resolution of the intussuscepted portion of the bowel. There was an 8 cm section of bruised bowel appreciated in the min-jejunal area, with areas of stenosis both proximal and distal to the bruised bowel. A decision was made to convert to open and 17 cm of small bowel was resected and an anastomosis was made. Histopathology of the resected bowel showed benign tissue with prominent serosal congestion, but otherwise unremarkable. The patient did well postoperatively, with a return of bowel function on postoperative day five.

## Discussion

Intussusception is a rare diagnosis in adults. Overall, 5% of intussusception diagnoses occur in adults, and only 1% of bowel obstructions in adults are associated with intussusception [3]. The etiology of an intussuscepted segment varies based on location within the gastrointestinal tract. A lead point is found in 80-90% of adult intussusception cases, and the remaining patients are characterized as having an idiopathic presentation [3]. When a lead point is found, the lead point is malignant in 60% of colonic intussusception and in 30% of small bowel intussusception [3]. The remaining 70% of small bowel intussusception can be attributed to benign causes such as an anatomic lead point (i.e., Meckel’s diverticulum) or a physiologic abnormality (i.e., abnormal peristalsis); if no physiologic or mechanical cause is identified, it is deemed idiopathic. Roughly only 8-20% of small bowel intussusception is idiopathic, and that number may be overestimated as benign etiologies may be underlying but left undiagnosed [3].

As apparent by the preceding statistics, the presentation of an idiopathic small bowel intussusception is exceedingly rare, and scarce research has been reported in the literature. Pain is the most common presenting symptom in adult intussusception, occurring in 71-90% of cases. Other common symptoms include hematochezia, nausea, vomiting, and a change in bowel habits [3]. The diagnostic modality most commonly used in recent years for intussusception has been abdominal CT. Studies have shown a 58-100% accuracy and 57-71% specificity in the diagnosis of intussusception with CT [4]. As apparent with this patient, a common finding on CT is the “target” sign.

Because of the high risk of malignancy historically attributed to adult intussusception, the decision to surgically resect the lesion has been the mainstay of treatment. There is no clear consensus on whether preoperative reduction of the intussusception in an adult patient should be attempted before undergoing surgical resection. A few case reports have documented the resolution of colonic intussusception post-colonoscopy or endoscopy [5-7]. Because of the possibility of perforation or seeding of cancerous cells, most surgeons decide to do without preoperative reduction unless clear evidence is available that the lead point is benign and not malignant [8]. In the case of entero-enteric intussusception, as in our patient, endoscopic reduction is not usually possible due to the inability of the scope to reach the intussuscepted segment, leaving surgery the mainstay of non-conservative management in these patients.

The decision to undergo surgical resection in adult intussusception has been challenged recently, as the frequency of an idiopathic etiology has increased in the past 10 years. A PubMed search with the term “adult idiopathic intussusception” and time range of “2010-2023” was conducted. A total of 22 cases of true adult idiopathic intussusception were found within that time range, a significant increase from the fewer than 10 reported cases in the years preceding 2010. A concise review of the aforementioned cases is presented in Table 1.

**Table 1:** Adult idiopathic intussusception case series review. M = male; F = female; I = entero-enteric; II = colo-colic; III = ileo-colic; IV = ileo-cecal; CT = computed tomography; SS = stool study; UGS = upper gastrointestinal series; sb = small bowel, XR Abd = abdominal X-ray; EGD = esophagogastroduodenoscopy; Lap = laparoscopy

Case number	Age	Sex	Segment	Imaging	Outcome	Symptom duration
1	32	M	I	CT	Lap	4 hours
2	34	F	I	CT, SS, UGS w/sb follow through	Conservative	24 hours
3	54	M	I	CT	Lap	Several hours
4	73	F	I	CT	Lap	2 months
5	30	M	I	CT	Lap	5 months, acute for two days
6	71	F	IV	CT	Lap	Several hours
7	54	F	I	CT	Lap	N/A
8	91	F	IV	CT	Endoscopic reduction	2 days
9	34	M	II	CT	Lap	Sudden
10	29	F	I	CT, XR Abd	Lap	N/A
11	53	F	II	CT, US	Colonoscopy reduced followed by lap 12 days later	N/A
12	25	F	IV	CT	Lap	24 hours
13	56	F	III	CT	High-pressure enema followed by colonoscopy	N/A
14	19	M	I	CT, EGD	Lap	2 days
15	22	F	I	CT	Lap	7 hours
16	46	F	II	CT	Lap	8 hours
17	26	M	I	CT	Lap	24 hours
18	21	F	I	CT	Lap	N/A
19	67	F	I	CT, barium	Lap	3 years
20	30	F	I	CT	Lap	N/A
21	34	M	I	CT	Conservative	N/A
22	32	M	I	CT	Lap	4 hours

The decision to opt for conservative management in patients who present with a definitive idiopathic presentation is still up for debate. From the few case reports that have been reported in the previous years, if small bowel intussusceptions without a malignant lead point are less than 3.5-3.8 cm, there is potentially a role for conservative management with serial examination and imaging to ensure resolution [2]. In a case report published by Thomas et al. [9], a patient who presented with a 3 cm jejunal-jejunal intussusception was worked up with the appropriate imaging, and no pathological lead point was found. The decision was made for the patient to undergo symptomatic management with antibiotics, antiemetics, and a nasogastric tube. The intussusception resolved on its own. Another case series described a similar occurrence in two patients where the authors labeled their jejunal-jejunal intussusception as “transient” because the intussusception resolved without surgery [10].

**Figure 2:**
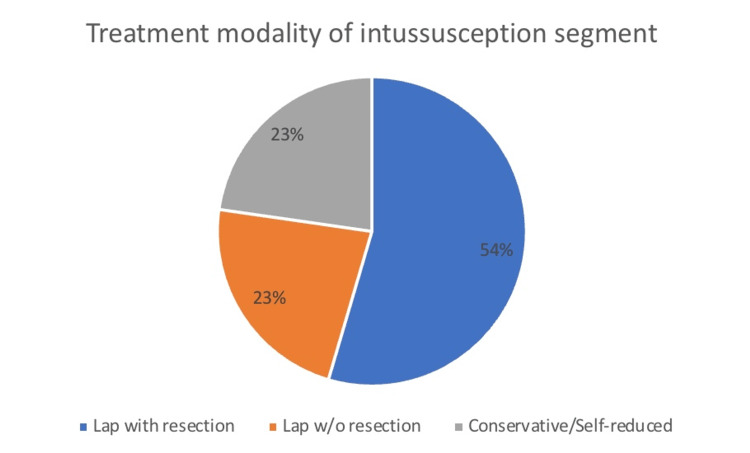
Treatment modalities for intussusception segment.

Nevertheless, according to the brief literature review conducted, most clinicians decided to opt for surgical resection without attention to the size of the intussusception. Figure 2 depicts that over three-quarters of small bowel idiopathic intussusceptions were surgically managed, with fewer than 25% conservatively managed.

**Figure 3:**
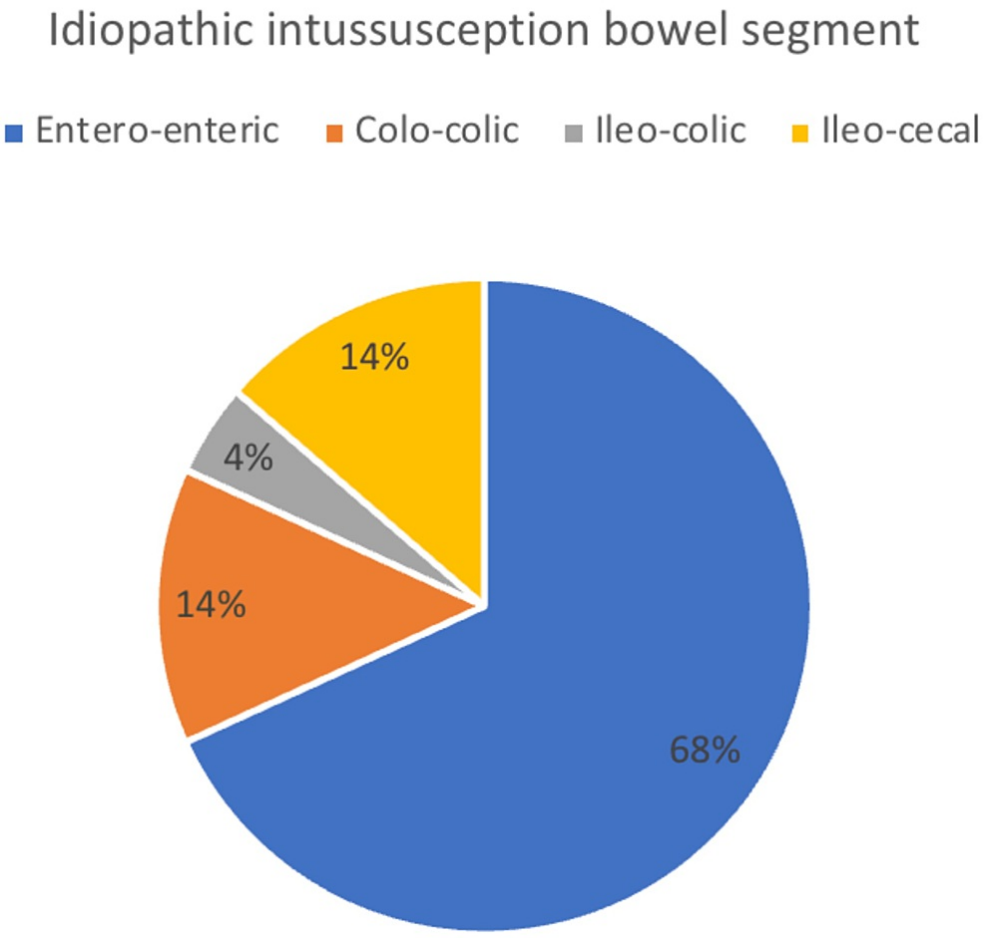
Idiopathic intussusception bowel segment.

Our case reported entero-enteric jejunal intussusception which seems to be the most common segment of bowel involved in non-pathologic adult intussusceptions. According to our brief literature review, over two-thirds of idiopathic adult intussusception discussed in the literature were entero-enteric at 68%, with the remaining 32% being categorized as colo-colic, ileo-cecal, and ileo-colic at 14%, 14%, and 4%, respectively (Figure 3). Based on this knowledge, we propose that small bowel intussusception may be able to be more conservatively managed. More research is needed on this topic, but we hope that our case report helps inform future management of this surgical issue.

## Conclusions

The patient in this case was found to have small bowel intussusception, with no lead point or evidence of malignancy, and was managed surgically with resection. This case report highlights the need for further studies comparing the management options of small bowel idiopathic intussusception in the adult population. Joint decision-making with each patient should be undertaken to ensure risks and benefits are discussed for both operative and non-operative management. We hope this case report highlights the need for more research and input into the management of this growing surgical issue.
